# YTHDC1 Regulates the Migration, Invasion, Proliferation, and Apoptosis of Rheumatoid Fibroblast-Like Synoviocytes

**DOI:** 10.3389/fimmu.2024.1440398

**Published:** 2024-10-29

**Authors:** Zhi-wei Feng, Chen-fei Yang, He-fang Xiao, Fa Yuan, Feng Chen, Bo Zhang, Jun Zhang, Min Tan, Ming-gang Guo

**Affiliations:** ^1^ Department of Orthopaedics, Nanchong Central Hospital, The Second Clinical Institute of North Sichuan Medical College, Nanchong, China; ^2^ Department of Orthopaedics, Lanzhou University Second Hospital, Lanzhou, China; ^3^ School of Nursing, North Sichuan Medical College, Nanchong, China; ^4^ Department of Rheumatology, Lanzhou University Second Hospital, Lanzhou, China

**Keywords:** YTHDC1, M^6^A modification, proliferation, apoptosis, rheumatoid arthritis

## Abstract

**Background:**

Rheumatoid arthritis (RA), a chronic autoimmune condition, is characterized by persistent synovial inflammation, bone degradation, and progressive joint deterioration. Despite considerable research efforts, the precise molecular mechanism underlying RA remains elusive. This investigation aims to elucidate the potential role and molecular mechanism of N6-methyladenosine (m^6^A) methylation regulators in the pathogenesis of RA.

**Methods:**

In this study, we employed bioinformatics tools to elucidate the association between RA and m^6^A modifications, aiming to identify potential biological markers. We extracted datasets GSE12021, GSE55235, and GSE55457 from the Gene Expression Omnibus (GEO) database for comprehensive analysis. Utilizing differential expression analysis, protein-protein interaction (PPI) analysis, and single-cell sequencing techniques, we identified pivotal hub genes implicated in the pathogenesis of RA. Subsequently, we assessed the correlation between these hub genes and the pathogenesis of RA using Gene Set Enrichment Analysis (GSEA). Both *in vivo* and *in vitro* experiments were performed to confirm the expression and functional roles of the identified key hub gene in RA.

**Results:**

Differential expression analysis, PPI analysis, and single-cell analysis identified three key hub genes (YTHDC1, YTHDC2, and YTHDF2) associated with RA. GSEA results further revealed that these genes are enriched in pathways associated with inflammatory responses. Subsequent correlation analysis demonstrated a significant negative correlation between YTHDC1 expression and CD8+ T cell levels. Notably, the gene and protein expression levels of YTHDC1 and YTHDF2 were significantly reduced in the synovial tissue of RA patients. Furthermore, silencing YTHDC1 in fibroblast-like synoviocytes (FLSs) significantly inhibited their migration, invasion, proliferation, and induced apoptosis.

**Conclusion:**

YTHDC1 may potentially be involved in the pathogenesis of RA through its regulation of migration, invasion, proliferation, and apoptosis in FLSs.

## 1 Introduction

Rheumatoid arthritis (RA) is characterized by chronic synovial inflammation and pronounced destruction of bone and cartilage within the affected joints (1–4). While the precise etiology of RA remains elusive, emerging research points to immune cell infiltration and phenotypic alterations in synoviocytes as central to the development of synovial inflammation (5). Fibroblast-like synoviocytes (FLSs) in RA exhibit neoplastic growth characteristics, with their marked and aberrant proliferation and apoptosis significantly contributing to synovial inflammation and bone destruction in RA (6–9). Regulating the balance between proliferation and apoptosis, coupled with managing inflammation and invasion of activated FLSs, presents a promising therapeutic approach for mitigating the severe progression of RA (10).

N6-methyladenosine (m^6^A) represents the most widespread modification of mRNA following transcription, intricately involved in various biological processes (11–13). M^6^A modification is involved in regulating various stages of mRNA metabolism, including mRNA expression (14, 15), translation efficiency (16–18), nuclear export (19, 20), splicing (14, 21, 22), and stability (13). Additionally, m^6^A modification is also widely involved in a variety of biological processes, such as development (22, 23), fertility (20), cell reprogramming (24–26), yeast meiosis (27), circadian period (19), embryonic stem cell self-renewal and differentiation (23, 24), osteoblast proliferation and differentiation (28), heat shock response (18), T cell homeostasis (29), and so on. The modification process involves a set of highly conserved enzymes, encompassing methyltransferases (writers), binding proteins (readers), and demethylases (erasers) (30, 31). Despite the association of m^6^A modification with a wide array of diseases, research focusing on m^6^A modification in RA remains insufficient.

FLSs are key players in the pathogenesis and progression of RA, contributing to synovial inflammation and joint damage (5). They promote RA by aggressively proliferating, invading tissues, secreting pro-inflammatory cytokines, forming pannus, and producing matrix metalloproteases (MMPs) that degrade cartilage (32, 33). Recent studies show a lower level of m^6^A modification in RA FLSs compared to those from healthy controls (34). Similarly, in collagen-induced arthritis (CIA), a model used to study RA, FLSs from affected rats also show reduced m6A levels (34). Research indicates that METTL3 enhances RA-FLSs activation and the inflammatory response by activating the nuclear factor kappa B pathway (35). Conversely, deleting METTL14 in a rat RA model reduces FLSs activation and lessens joint damage (36). Additionally, inhibiting ALKBH5 decreases the proliferation, migration, and invasion of RA FLSs (34). Although these findings are significant, the comprehensive data on the role of m^6^A modification in RA remains incomplete, necessitating further studies to elucidate its impact.

In this investigation, our bioinformatics analysis revealed that YTHDC1 is intricately associated with the development of synovial inflammation in RA. When comparing synovial tissues from RA patients to trauma controls (TC), we observed markedly reduced expression levels of YTHDC1. Further experiments showed that overexpressing YTHDC1 significantly inhibits the migration, invasion, and proliferation of RA-FLSs, while also promoting apoptosis. These findings suggest that YTHDC1 plays a critical role in fibroblast behaviors that contribute to organ fibrosis. Accordingly, our study advances the understanding of m^6^A-driven mechanisms in dysregulated fibroblast-related disorders.

## 2 Materials and methods

### 2.1 Data processing and differential expression analysis

RA microarray datasets were obtained from the GEO database by filtering for “Expression profiling by array”, “Homo sapiens”, and “sample count >20”. Three gene expression profiles (GSE12021, GSE55457, and GSE55235) were chosen for further analysis from the GPL96 platform.

The dataset was downloaded in TXT format from the GEO database. Batch effects were eliminated and the data was standardized with the “SVA” package (37). The “Limma” package (38) was used to identify differentially expressed genes (DEGs). To identify DEGs, set the criteria to “*P*-value < 0.05 and |logFC| > 1”.

### 2.2 Immune infiltration analysis

We conducted an analysis of immune cell infiltration within RA datasets in comparison to samples from healthy individuals, employing the computational tool CIBERSORT (39). The parameter “PERM” was set to 1000, with a significance threshold established at a *P*-value of < 0.05. Subsequently, we computed the relative proportions of distinct immune cell subsets, presenting the findings through graphical representations in the form of bar charts. Heatmaps depicting the distribution of the 22 identified immune cell types were generated utilizing the “pheatmap” package, while their relative abundance was depicted using the “vioplot” package. Furthermore, a correlation heatmap elucidating the interrelations among the aforementioned 22 types of infiltrating immune cells was constructed employing the “corrplot” package.

### 2.3 Functional enrichment analysis of DEGs

To investigate the roles and pathways associated with DEGs, we employed the “clusterProfiler” package (40) to conduct Gene Ontology (GO) (41) and Kyoto Encyclopedia of Genes and Genomes (KEGG) (42) pathway enrichment analyses. We established statistical significance at the *p*-value and *q*-value thresholds of less than 0.05. Subsequently, Gene Set Enrichment Analysis (GSEA) (43) was conducted using the same “clusterProfiler” R package, focusing on the immunologic signature gene set (C2 gene sets) sourced from the Molecular Signatures Database (MSigDB, https://www.gsea-msigdb.org/).

### 2.4 Investigation and evaluation of m^6^A regulators

For further investigation, we selected twenty-three m^6^A regulators widely recognized in the literature, including eight m^6^A writers, two m^6^A erasers, and thirteen m^6^A readers (**Table 1**). To examine their potential interactions, we used the STRING website (https://string-db.org/) (44) to construct a protein-protein interaction (PPI) network (45), setting the confidence score at 0.4 and the maximum number of interactors at 0. Subsequently, we employed the Molecular Complex Detection (MCODE) (46) and Cytohubba (47) plugins in Cytoscape (v.3.8.2) (48) with default parameters to identify hub genes within this network.

**Table 1:** The description of 20 m^6^A RNA methylation regulators.

Gene	Gene ID	Type	Gene	Gene ID	Type	Gene	Gene ID	Type
METTL3	56339	Writers	FTO	79068	Erasers	FMR1	2332	Readers
METTL14	57721	Writers	ALKBH5	54890	Erasers	LRPPRC	10128	Readers
WTAP	9589	Writers	YTHDC1	91746	Readers	HNRNPA2B1	3181	Readers
RBM15	64783	Writers	YTHDC2	64848	Readers	IGFBP1	3484	Readers
RBM15B	29890	Writers	YTHDF1	54915	Readers	IGFBP2	3485	Readers
ZC3H13	23091	Writers	YTHDF2	51441	Readers	IGFBP3	3486	Readers
METTL16	79066	Writers	YTHDF3	253943	Readers	RBMX	27316	Readers
VIRMA	25962	Writers	HNRNPC	3183	Readers			

### 2.5 Single cell data analysis

Additional analyses were performed on the RA single-cell dataset GSE159117, which comprised one sample diagnosed with RA. The expression levels of genes in each cell batch were systematically quantified. The number of genes detected in each cell must fall within a range of two to half of the average gene count observed in the same batch. Additionally, the proportion of mitochondrial genes should not exceed a specific threshold (typically 5%-10%) to exclude cells that might be stressed or dead. Genes expressed in fewer than 0.1% of cells are also excluded. Quality control assessments confirmed that all cells within this dataset adhered to established standards. Principal component analysis (PCA) was executed using the “Seurat” package, applying the JackStraw and PCElbowPlot functions to ascertain significant principal components (PCs). The FindAllMarkers function of Seurat was utilized to delineate specific genes characteristic of each cell subpopulation. Subsequently, cell clustering and the visual representation of clusters via UMAP were conducted using the RunUMAP function. Marker genes were annotated using the “singleR” package and were further refined with CellMarker adjustments based on gene characteristics.

The “Seurat” and “DoubletFinder” packages facilitated the filtration of cells and genes. Cells were only retained for subsequent analyses if they met the following quality control parameters: (i) the number of detected genes per cell was no more than twice and no less than half of the mean gene count observed in cells from the same sample; (ii) mitochondrial gene expression constituted less than 20% of the total gene count per cell; (iii) cells passed the standard doublet removal protocol of the DoubletFinder package. After cell and gene filtration, samples were integrated using the Harmony software package and clustered unsupervised. DEGs within each cluster were identified using the FindMarkers function of Seurat.

### 2.6 Correlation analysis of hub genes and the immune status

Spearman’s rank correlation analysis was performed using the R package “ggcorrplot” to demonstrate the connections between the expression levels of hub genes and the immune status.

### 2.7 Isolation and culture of primary FLSs

Synovial tissue was harvested from RA patients undergoing knee arthroplasty or synovial resection at the Second Hospital of Lanzhou University. Control samples were obtained from trauma patients diagnosed with meniscal or cruciate ligament injury. Exclusion criteria included concurrent medical conditions and any history of tobacco or alcohol use. Ethical approval for the study was granted by the Medical Ethics Committee of the Second Hospital of Lanzhou University, China, and informed consent was obtained from all participants in writing.

Tissue specimens were finely chopped into pieces measuring 2–3 mm³ and subjected to enzymatic digestion using 4 mg/mL collagenase II for one hour. The reaction was terminated by the addition of high-glucose DMEM supplemented with 10% fetal bovine serum (FBS) and 1% penicillin/streptomycin. Following this, the suspension was centrifuged at 1200 rpm for five minutes and the pellet was resuspended and maintained in DMEM containing 10% FBS for subsequent analyses.

### 2.8 Transient transfection

To achieve transient knockdown of YTHDC1 in fibroblast-like synoviocytes (FLSs), the cells were transfected with small interfering RNA (siRNA) procured from GenePharma (Shanghai, China). The specific sequences of the targeted siRNA are provided in **Table 2**. Furthermore, a lentiviral plasmid designed to overexpress YTHDC1 (oe-YTHDC1) was synthesized by GenePharma, with an empty plasmid serving as the control transfection (oe-NC).

**Table 2:** siRNA targeting sequences.

Gene	siRNA targeting sequences
Si-NC	sense 5’- UUCUCCGAACGUGUCACGUTT -3’
	antisense 5’- ACGUCACACGUUCGGAGAATT -3’
si1-YTHDC1	sense 5’- UGCCUCCAGAGAACCUUAUAA -3’
	antisense 5’- UUAUAAGGUUCUCUGGAGGCA -3’
si2-YTHDC1	sense 5’- GTCGACCAGAAGATTATGATA -3’
	antisense 5’- CTAGCTGGTCTTCTAATACTA -3’
si3-YTHDC1	sense 5’- ATCGAGTATGCAAATATTGAA -3’
	antisense 5’- TAGCTCATACTTGTTATAACT -3’

### 2.9 RNA m^6^A measurement

Per the manufacturer’s guidelines, m^6^A levels were measured with an EpiQuik m^6^A RNA Methylation Quantification Kit (Colorimetric) (Catalog# P-9005, AmyJet Scientific, Wuhan, China). RNA was extracted from synovial tissues, and aliquots of 200 ng were utilized to determine m6A levels.

### 2.10 Flow cytometric analysis

To assess cell apoptosis, RA-FLSs were treated with 5 µL of Annexin V-APC and 10 µL of 7-Aminoactinomycin D (7-AAD) solution (MultiSciences, Hangzhou, China) for 5 minutes at room temperature in a light-protected environment. Alternatively, cells were incubated with 5 µL of Annexin V-FITC and 10 µL of Propidium Iodide (PI) Staining Solution (Yeasen, Shanghai, China) under similar conditions for 10 minutes. Apoptotic cells were quantified using a CytoFLEX flow cytometer (Beckman, Brea, California, USA), and data were analyzed with CytExpert 2.4 software (Beckman, Brea, California, USA).

### 2.11 TUNEL staining

RA-FLSs were cultured in 24-well plates at a density of 8 × 10^4^ cells per well. After 24 h of incubation, the cells were fixed with 4% paraformaldehyde (PFA) for 30 min at room temperature. Subsequent to fixation, the cells underwent staining using a terminal deoxynucleotidyl transferase-mediated dUTP nick-end labeling (TUNEL) assay kit (Yeasen, Shanghai, China). The nuclei were counterstained with 4′,6-diamidino-2-phenylindole (DAPI, Beyotime, Shanghai, China) at 37°C for 5 min. Apoptotic cells were then visualized under an inverted fluorescence microscope (IX53, Olympus, Tokyo, Japan), displaying green fluorescence for apoptotic markers and blue for the nuclei.

### 2.12 EdU staining

Cell proliferation was assessed using the BeyoClick™ EdU Kit (Beyotime, Shanghai, China), following the manufacturer’s instructions. Cells were initially washed thrice with PBS, each wash lasting 4 min, and subsequently fixed with 4% paraformaldehyde for 30 min. Permeabilization was achieved by treating the cells with 0.3% Triton X-100. The cells were then stained with the provided reaction solution. After staining, the cells were incubated with Hoechst stain for 5 min to label the nuclei. Images were captured using a fluorescence microscope (Olympus IX73, Tokyo, Japan) equipped with a 40x and 60x oil immersion objective.

### 2.13 Cell migration and scratch assay

FLSs were seeded at a density of 2 × 10^5^ cells per well in six-well plates and incubated in DMEM supplemented with 10% FBS at 37°C for 24 h. Upon reaching 100% confluence, the cell monolayer was deliberately wounded using a 200 µL pipette tip. Subsequently, the wells were rinsed twice with PBS to eliminate non-adherent cells. The cells were then maintained in serum-free DMEM at 37°C. The gap created by the scratch was imaged at 0 and 24 h post-wounding using a light microscope to assess wound closure.

### 2.14 Cell invasion and transwell experiment

Cell migration was evaluated using a Transwell chamber featuring an 8.0 µm pore size and a 6.5 mm diameter (Corning, NY, USA). FLSs were seeded at a density of 6 × 10^4^ cells per well in 100 μl of serum-free DMEM in the upper chamber, while the lower chamber was filled with DMEM supplemented with 10% FBS, serving as a chemoattractant. Following incubation at 37°C for 24 h, cells remaining on the upper surface of the membrane were removed using a cotton swab. Cells that had migrated to the lower surface of the membrane were fixed with methanol for 30 min, stained with 0.1% crystal violet for 30 min, and then visualized under an optical microscope. The number of cells that traversed the membrane was quantified by counting the stained cells in five randomly selected microscopic fields. For the invasion assays, Transwell chambers were pre-coated with a Matrigel matrix (BioZellen, NE, USA).

### 2.15 RNA extraction and quantitative real-time PCR

Total RNA was isolated from synovial tissue and FLSs utilizing Trizol reagent (Invitrogen, Carlsbad, CA, USA). The RNA was subsequently reverse transcribed into complementary DNA (cDNA) using the PrimeScript RT reagent with gDNA Eraser kit (Takara Bio, Inc., Kyoto Prefecture, Japan). Quantitative real-time PCR (qRT-PCR) assays were conducted using SYBR Green qPCR Mix (Takara Bio, Inc., Japan) on a CFX96 quantitative PCR system (Bole, Inc., California, USA). The expression levels of target genes were normalized to GAPDH and quantified employing the 2^-ΔΔCq^ method (49). Primer sequences for qRT-PCR are provided in **Table 3**.

**Table 3:** The sequence of primers.

Gene	Forward and reverse primer
YTHDC1	F:5’ TCAGGAGTTCGCCGAGATGTGT 3’
	R:5’ AGGATGGTGTGGAGGTTGTTCC 3’
YTHDC2	F:5’ TGCCTTTGCTCAGGTCTTTC 3’
	R:5’ CCAGCCATTTGATGCTTTAC 3’
YTHDF2	F:5’ AGCCCCACTTCCTACCAGATG 3’
	R:5’ TGAGAACTGTTATTTCCCCATGC 3’
GAPDH	F:5’ TGTGTCCGTCGTGGATCTGA 3’ R:5’ TTGCTGTTGAAGTCGCAGGAG 3’

### 2.16 Western blot

Total protein was extracted from synovial tissue and FLSs. A quantity of 30 µg protein per lane was resolved by 10% SDS-PAGE and subsequently transferred to PVDF membranes (Immobilon-P, Merck, Germany). The membranes were blocked with 5% Bovine Serum Albumin (BSA) at room temperature for one hour. Overnight incubation at 4°C with primary antibodies was followed by washes in TBST and a one-hour incubation with secondary antibodies at room temperature. Protein bands were detected using a hypersensitive ECL chemiluminescence solution (Beyotime, Shanghai, China) and imaged with a MiniChemi 610 plus chemiluminescence fluorescence analysis system (Sagecreation, Beijing, China). Protein band densities were quantified using Image J software. The antibodies utilized in this study comprised YTHDC1 (Abcam, ab259990, Monoclonal), YTHDC2 (Abcam, ab220160, Monoclonal), YTHDF2 (Abcam, ab220163, Monoclonal), along with PCNA (Proteintech, 10205-2-AP, Polyclonal), CDK4 (Proteintech, 11026-1-AP, Polyclonal), Cyclin D1 (Proteintech, 60186-1-Ig, Monoclonal), Bcl-2 (Proteintech, 60178-1-Ig, Monoclonal), Bax (Proteintech, 50599-2-Ig, Polyclonal), Cleaved-caspase-3 (Proteintech, 25128-1-AP, Polyclonal), and β-actin (Proteintech, 20536-1-AP, Polyclonal).

### 2.17 Immunofluorescence

Initially, specimens were fixed in a 4% paraformaldehyde solution overnight, followed by decalcification in a 10% EDTA solution. They were then embedded in paraffin, and sectioned into 4 µm thick slices. The sections were blocked with 5% bovine serum albumin for one hour. Overnight incubation at 4°C was carried out with primary antibodies: anti-VIMENTIN (Abcam, 1:1000), anti-YTHDC1 (Abcam, 1:500), and anti-YTHDF2 (Abcam, 1:1000). After this, sections were incubated with a fluorescent secondary antibody (1:500, Proteintech) for one hour in the dark. Nuclei were stained using DAPI (Biosharp, Hefei, China), and images were captured using the PerkinElmer Operetta CLS (PerkinElmer, Waltham, USA).

### 2.18 Histological and immunohistochemical analysis

Sections were stained using Hematoxylin and Eosin (H&E, Solarbio, Beijing, China). IHC staining was conducted with the UltraSensitive™ IHC Kit (MXB Biotechnologies, Shanghai, China), utilizing primary antibodies anti-YTHDC1 (Abcam, 1:500) and anti-YTHDF2 (Abcam, 1:1000). Fluorescence microscopy (Olympus, Japan) was employed to capture the images.

### 2.19 Statistical analysis

The statistical analysis was performed utilizing Prism 8.0 (GraphPad Software, Inc., San Diego, California, USA). Comparisons between two groups were made, adhering to a normal distribution, and analyzed via t-tests. Multigroup comparisons were performed utilizing tests for variance homogeneity and one-way ANOVA. The results are presented as mean ± standard deviation, with statistical significance defined as *P*-value < 0.05. All experiments were systematically conducted in triplicate to ensure both robustness and reproducibility. The mechanism schematic was illustrated using Figdraw (www.figdraw.com).

## 3 Results

### 3.1 Identification of DEGS in RA

**Figure 1:**
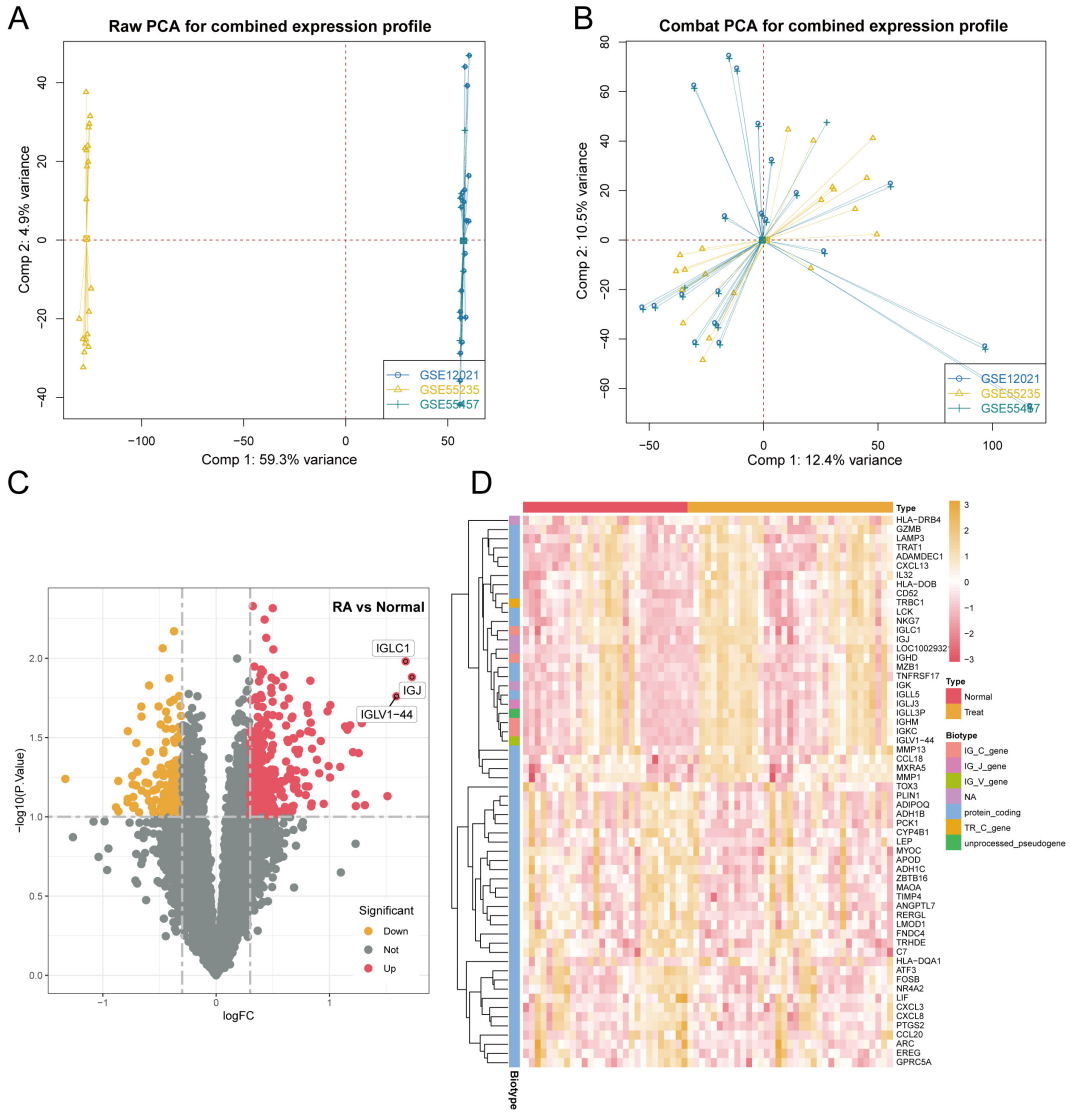
Identification of DEGs in synovial tissue from patients with RA included: **(A)** Results of principal component analysis (PCA) before batch effect removal. **(B)** PCA results following batch effect removal. **(C)** Volcano plot representing all normalized mRNA expression data. **(D)** Heatmap illustrating DEGs in synovial tissue from RA patients, with criteria of |log FC| > 1 and *P*-value < 0.05.

Three RA microarray datasets (GSE12021, GSE55457, and GSE55235) were obtained from the GEO database for analysis. These datasets were generated from RA patients and healthy controls’ synovial tissue samples using Affymetrix Human Genome U133A arrays (GPL96 platform). The integration of the three datasets was performed using the SVA method, the results of which are depicted in **Figure 1A** through a PCA plot of the original datasets. Subsequently, **Figure 1B** shows the merged data after de-batching, demonstrating effective integration. Differential expression analysis was subsequently carried out on the integrated datasets, comparing synovial tissues from RA patients and healthy controls, resulting in the identification of 5,714 DEGs based on a significance threshold of *P*-value < 0.05 and |logFC| > 1. The distribution of these DEGs is illustrated in **Figures 1C, D** through volcano plot and heat map, respectively.

### 3.2 Functional enrichment analyses of DEGs and immune infiltration analysis

**Figure 2:**
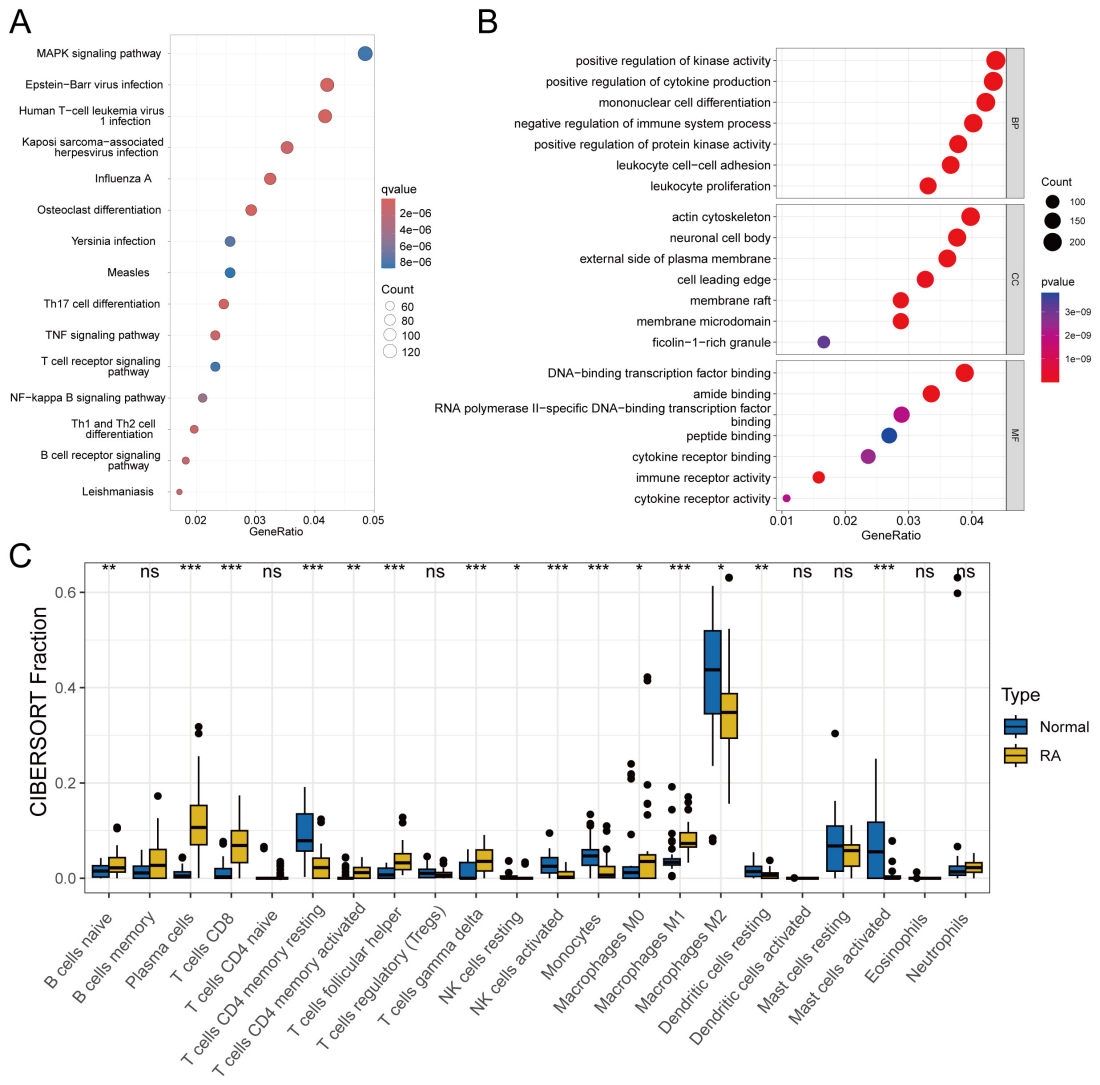
Enrichment analysis and immune infiltration analysis included: **(A)** KEGG enrichment analysis of DEGs with a q-value < 0.05. **(B)** GO enrichment analysis of DEGs with a *P*-value < 0.05. **(C)** Analysis of differences in immune cell infiltration, with significance defined as *P*-value < 0.05. statistical significance denoted as ns (not significant), *P < 0.05, **P < 0.01, ***P < 0.001.

**Figure 2A** displays the results of KEGG enrichment analysis for DEGs, revealing enrichment in pathways such as Epstein-Barr virus infection and Human T-cell leukemia virus1 infection. Concurrently, **Figure 2B** illustrates the GO enrichment results, indicating predominant enrichment in pathways related to mononuclear cell differentiation, membrane microdomain, and immune receptor activity. Subsequently, in **Figure 2C**, we conducted immune infiltration analysis to compare immune responses between the normal and disease groups. This analysis revealed significant differences in various immune cell populations, including CD8+ T cells and resting dendritic cells.

### 3.3 Single cell analysis

**Figure 3:**
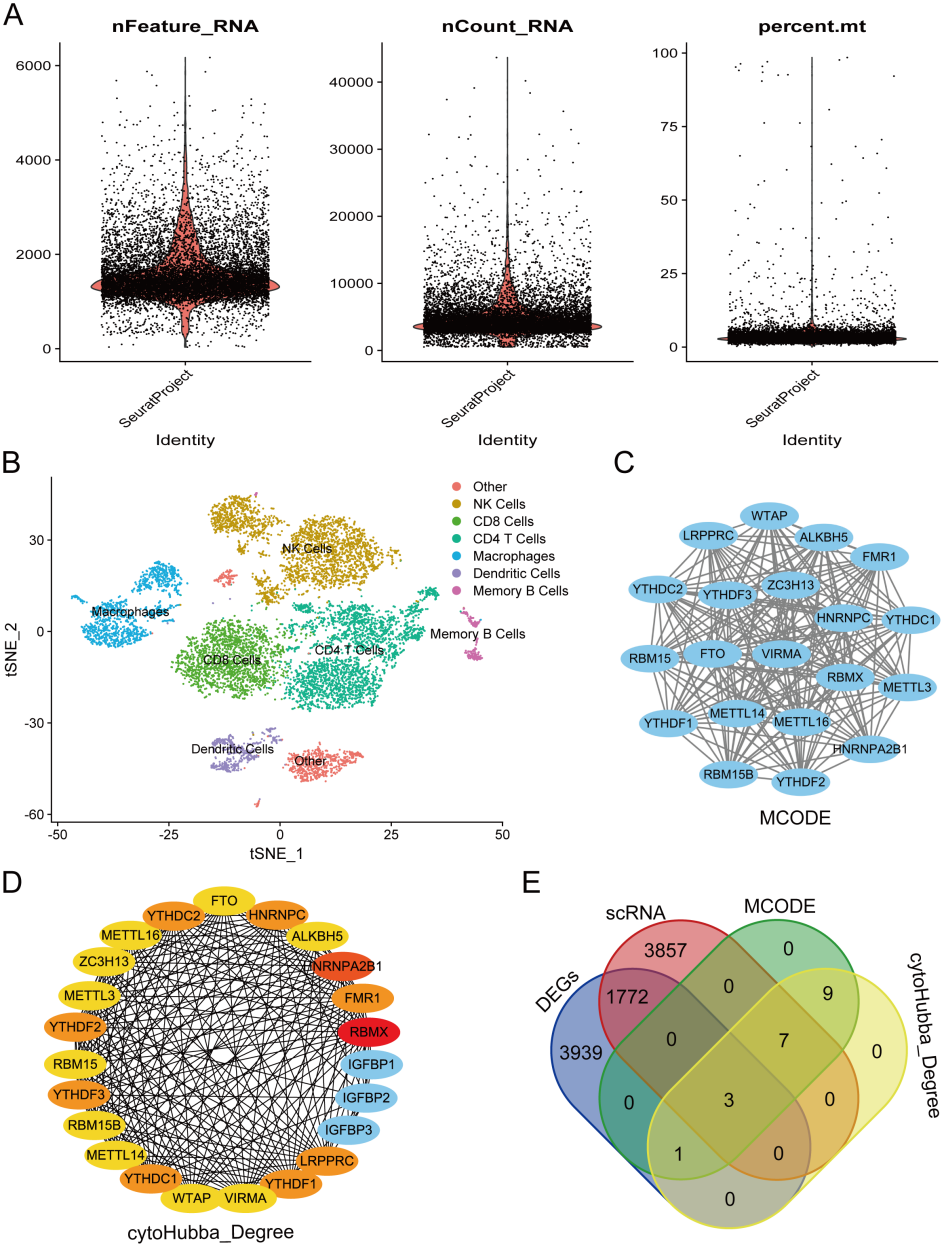
Screening of hub genes involved the following steps: **(A)** Quality control chart showing single-cell data before filtering, including all cells to provide a full overview of the dataset. **(B)** Results from cluster analysis of single-cell data. **(C)** Hub genes were identified from the PPI network using the MCODE algorithm. **(D)** Hub genes were extracted from the PPI network via the Degree algorithm. **(E)** Venn diagram illustrating the overlap of key genes.

From the single-cell dataset, we identified seven distinct clusters: Other, NK Cells, CD8+ T Cells, CD4+ T Cells, Macrophages, Dendritic Cells, and Memory B Cells. The data shown in **Figure 3A** are the original data before filtering, and **Figure 3B** presents the cluster analysis results using t-SNE. RA is linked to the activation of T cells and immune cells, leading to joint synovitis (50). Specifically, in RA, dendritic cells (DCs) are thought to present arthritogenic antigens to T cells (51). Abatacept, a potent T-cell activation inhibitor, is approved for treating RA, juvenile idiopathic arthritis, and psoriatic arthritis (52). Given the significance of T cells in RA, we focused on the CD8+ T Cells and Dendritic cell clusters, identifying 5,638 intersecting genes (**Supplementary Table 1**). We then constructed a PPI network of 23 m^6^A regulators using the STRING database. Using MCODE and cytoHubba’s Degree algorithms, we identified 20 hub genes from this network (**Figures 3C, D**), with three common genes—YTHDC2, YTHDF2, and YTHDC1—emerging as key regulators (**Figure 3E**).

### 3.4 GSEA analysis and correlation analysis of key genes

**Figure 4:**
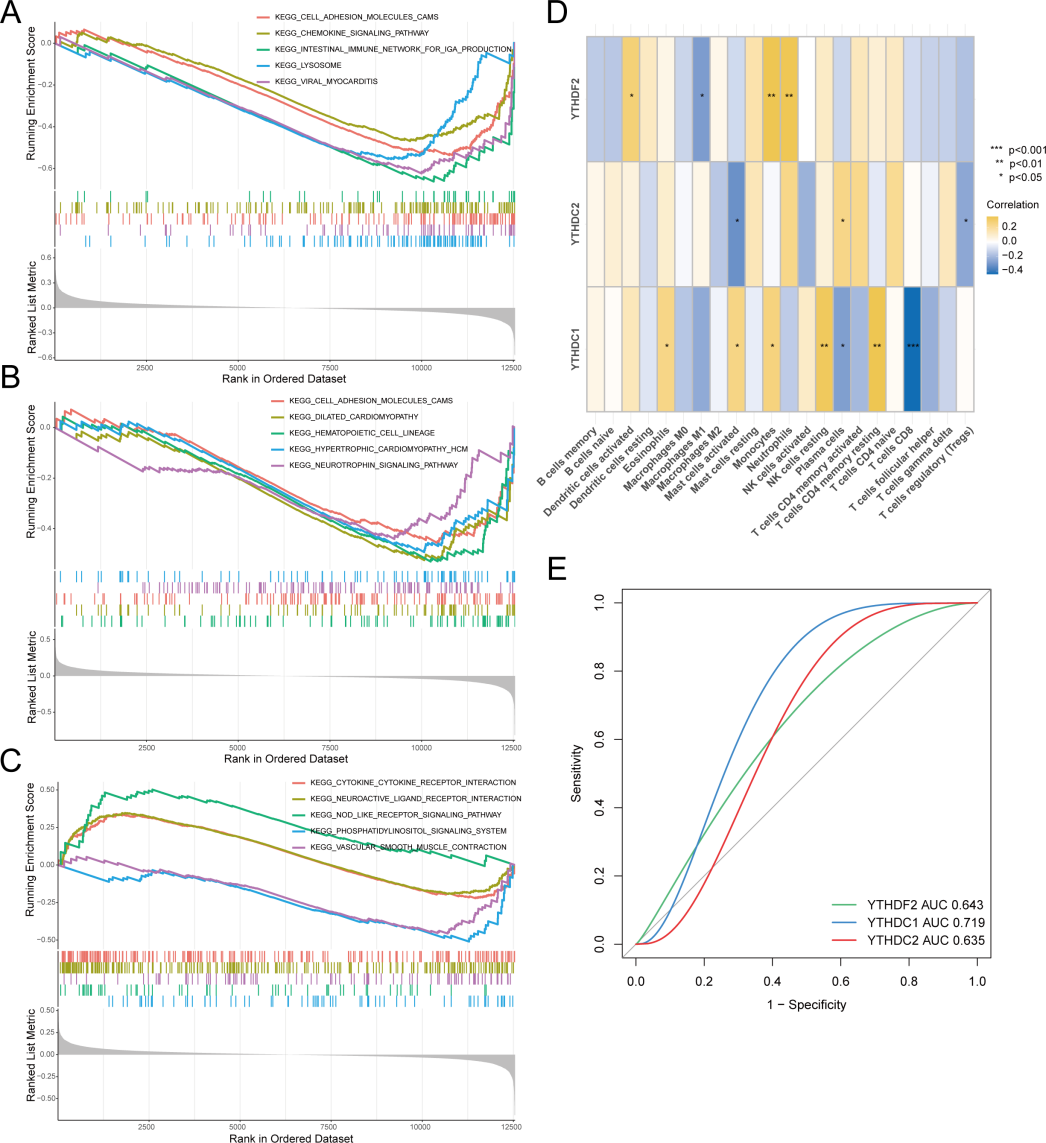
Correlation analysis between hub genes and RA. Gene Set Enrichment Analysis (GSEA) included: **(A)** Pathway enrichment analysis for YTHDC1. **(B)** Pathway enrichment analysis for YTHDC2. **(C)** Pathway enrichment analysis for YTHDF2. **(D)** Analysis of the correlation between key genes and immune cell infiltration. **(E)** Hub genes in the merged dataset were analyzed using ROC curves.

We performed GSEA analysis individually on three genes. **Figure 4A** illustrates the enrichment results for YTHDC1, notably in pathways including the cell adhesion molecule and chemokine signaling pathways. **Figure 4B** reveals that YTHDC2 is enriched in pathways such as the cell adhesion molecule and hematopoietic cell lineage pathways. Furthermore, **Figure 4C** presents the enrichment analysis for YTHDF2, demonstrating enrichment in pathways such as the chemokine receptor interaction and hematopoietic cell lineage pathways.

Moving forward, we executed a correlation analysis between immune infiltration and key genes, as depicted in **Figure 4D**. Notably, YTHDC1 exhibited correlations with various immune cells, particularly showing a significant negative correlation with CD8+ T cells, indicative of a potential role in the pathogenesis of RA. To investigate the diagnostic efficiency of the three hub genes, we conducted a ROC curve analysis, with hub genes having an AUC value > 0.7 serving as diagnostic markers. The AUC values in the merged dataset were 0.643 for YTHDF2, 0.719 for YTHDC1, and 0.635 for YTHDC2 (**Figure 4E**).

### 3.5 Validation of the hub genes expression in synovium from patients with RA

**Figure 5:**
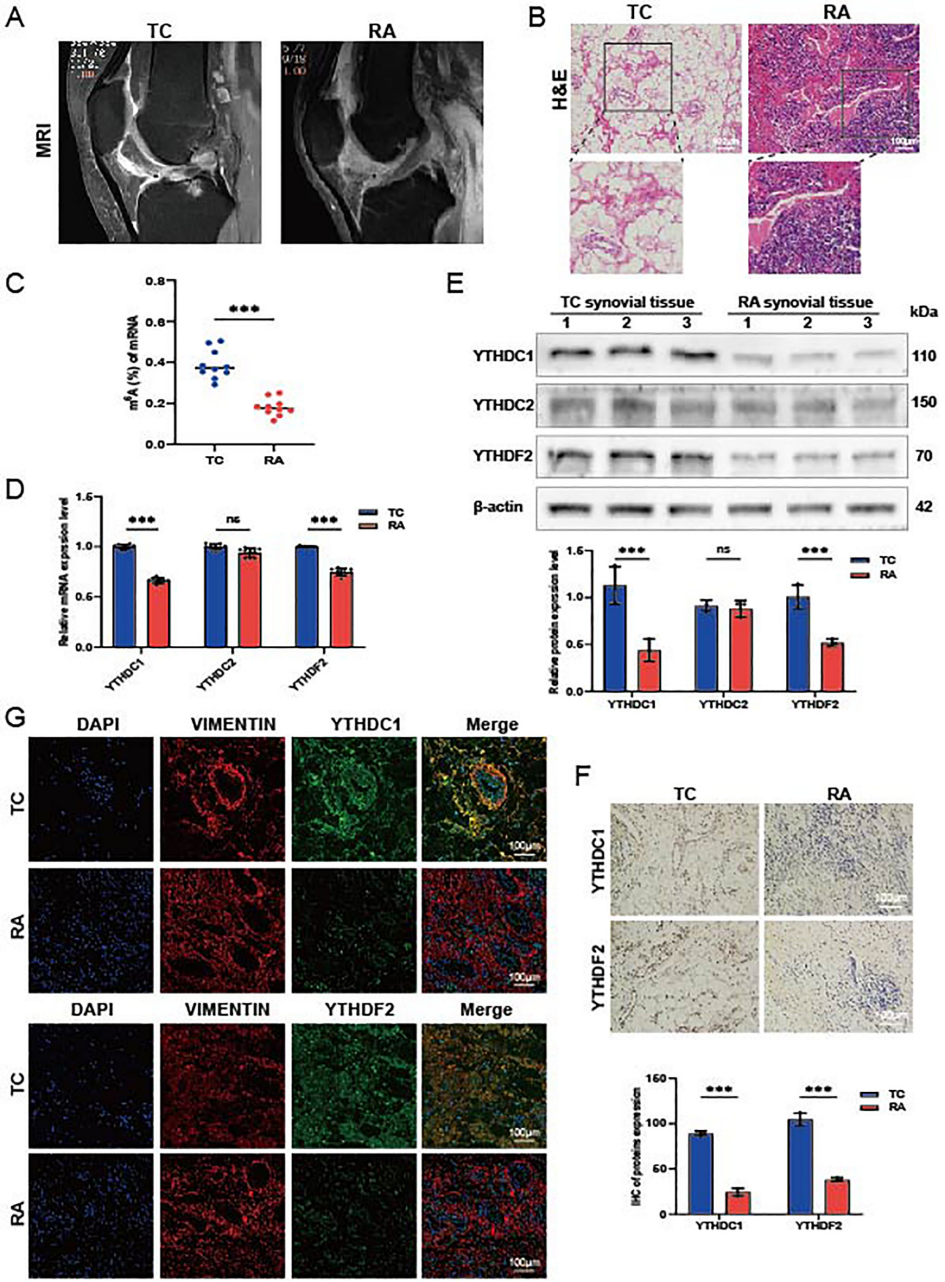
Expression of hub genes in synovial tissue from patients with RA. **(A)** Representative MRI scans. **(B)** Histological evaluations of H&E-stained synovial tissue sections (n = 3). **(C)** m^6^A modification levels quantified via qRT-PCR, normalized against GAPDH (n = 10). **(D)** mRNA expression levels of YTHDC1, YTHDC2, and YTHDF2 in TC and RA samples (n = 9). **(E)** Protein concentrations of YTHDC1, YTHDC2, and YTHDF2 in TC and RA samples (n = 3). **(F)** IHC images showing the expression of YTHDC1 and YTHDF2 in TC and RA samples (n = 3). **(G)** IF staining displaying YTHDC1 and YTHDF2 in TC and RA samples, with DAPI (blue), VIMENTIN (red), YTHDC1/YTHDF2 (green), and merged images (n = 3). Data are presented as means ± SD; significance levels are indicated as ns (not significant), ***P < 0.001.

Synovial tissues were collected from ten patients with RA and ten trauma control subjects (TC). Magnetic resonance imaging (MRI) analysis indicated thickening of the RA synovium, unlike that observed in the TC group (**Figure 5A**). H&E staining of the RA synovial tissues revealed increased cellular density and heterogeneity within the intercellular matrix. Notably, there was a marked increase in both the number and staining intensity of cell nuclei, indicative of heightened cell proliferation and extensive infiltration by inflammatory cells (**Figure 5B**). We measured m6A modification levels in RA synovial tissues using a colorimetric RNA m6A quantification method. The results demonstrated diminished m6A levels in RA synovium compared to TC synovium (**Figure 5C**). Quantitative real-time PCR (QRT-PCR) and Western blot analyses revealed a reduction in YTHDC1 and YTHDF2 expression in the RA synovium; however, the reduction in YTHDC2 expression was not statistically significant (**Figures 5D, E**). IHC and IF analyses further confirmed the downregulation of YTHDC1 and YTHDF2 in the RA group relative to the TC, as evidenced by diminished fluorescence intensity (**Figures 5F, G**). These findings collectively suggest significant reductions in YTHDC1 and YTHDF2 levels in RA synovium.

### 3.6 YTHDC1 overexpression promotes apoptosis of RA-FLSs

**Figure 6:**
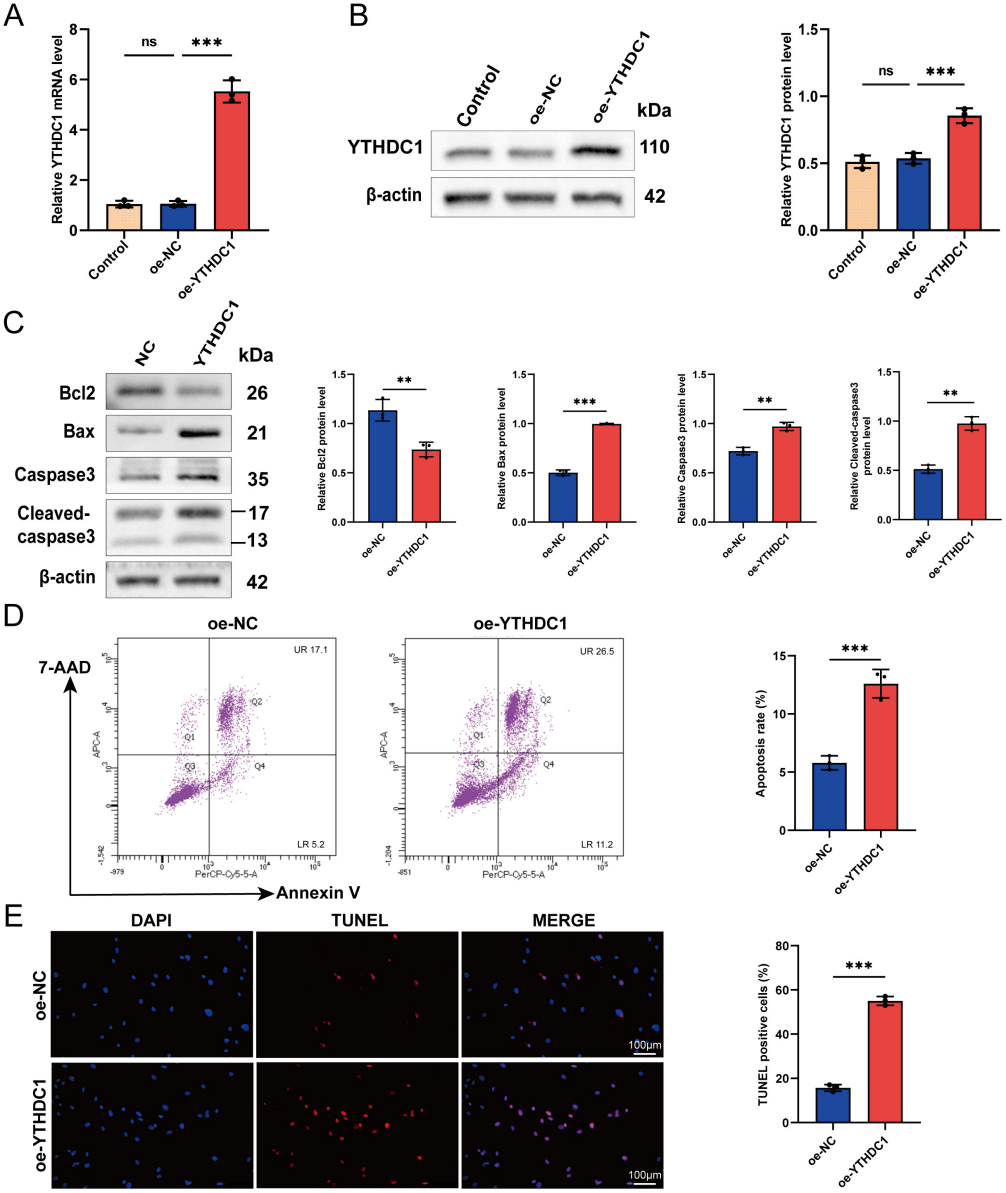
YTHDC1 modulates apoptosis in RA-FLSs. **(A, B)** Overexpression of YTHDC1 in RA-FLSs was performed, with efficacy confirmed via **(A)** quantitative RT-PCR and **(B)** Western blot analysis. **(C)** Western blotting assessed the protein levels of Bcl-2, Bax, Caspase-3, and Cleaved-caspase-3 subsequent to YTHDC1 overexpression. **(D)** Apoptosis in RA-FLSs following YTHDC1 overexpression was quantified using flow cytometry with 7-AAD and Annexin V staining (n = 3). **(E)** Apoptotic cells were further evaluated by TUNEL staining, and the number of TUNEL-positive cells was quantified (n = 3). Data are presented as means ± SD; significance levels are indicated as ns (not significant), **P < 0.01, ***P < 0.001.

Synovial tissue samples were procured from two patients diagnosed with RA for this study. FLSs were meticulously isolated from the synovial tissues of these RA patients and subsequently utilized in all downstream functional assays. Through lentiviral vector transfection (oe-YTHDC1), we increased the expression of YTHDC1 in RA-FLSs. QRT-PCR and Western blot analysis verified a marked increase in YTHDC1 expression in the overexpression group (**Figures 6A, B**). In exploring YTHDC1’s role in the apoptosis of RA-FLSs, we noted that YTHDC1 overexpression led to augmented apoptosis, evidenced by elevated levels of the pro-apoptotic proteins Bax, Caspase3, and Cleaved-caspase3, and a decline in the anti-apoptotic protein Bcl-2 (**Figure 6C**). Moreover, there was a significant rise in both early and late apoptotic cell populations (**Figure 6D**), as well as an increase in TUNEL-positive cells (**Figure 6E**). These observations together affirm that YTHDC1 overexpression facilitates apoptosis in RA-FLSs.

### 3.7 YTHDC1 overexpression impairs RA-FLSs migration, invasion, and proliferation *in vitro*

**Figure 7:**
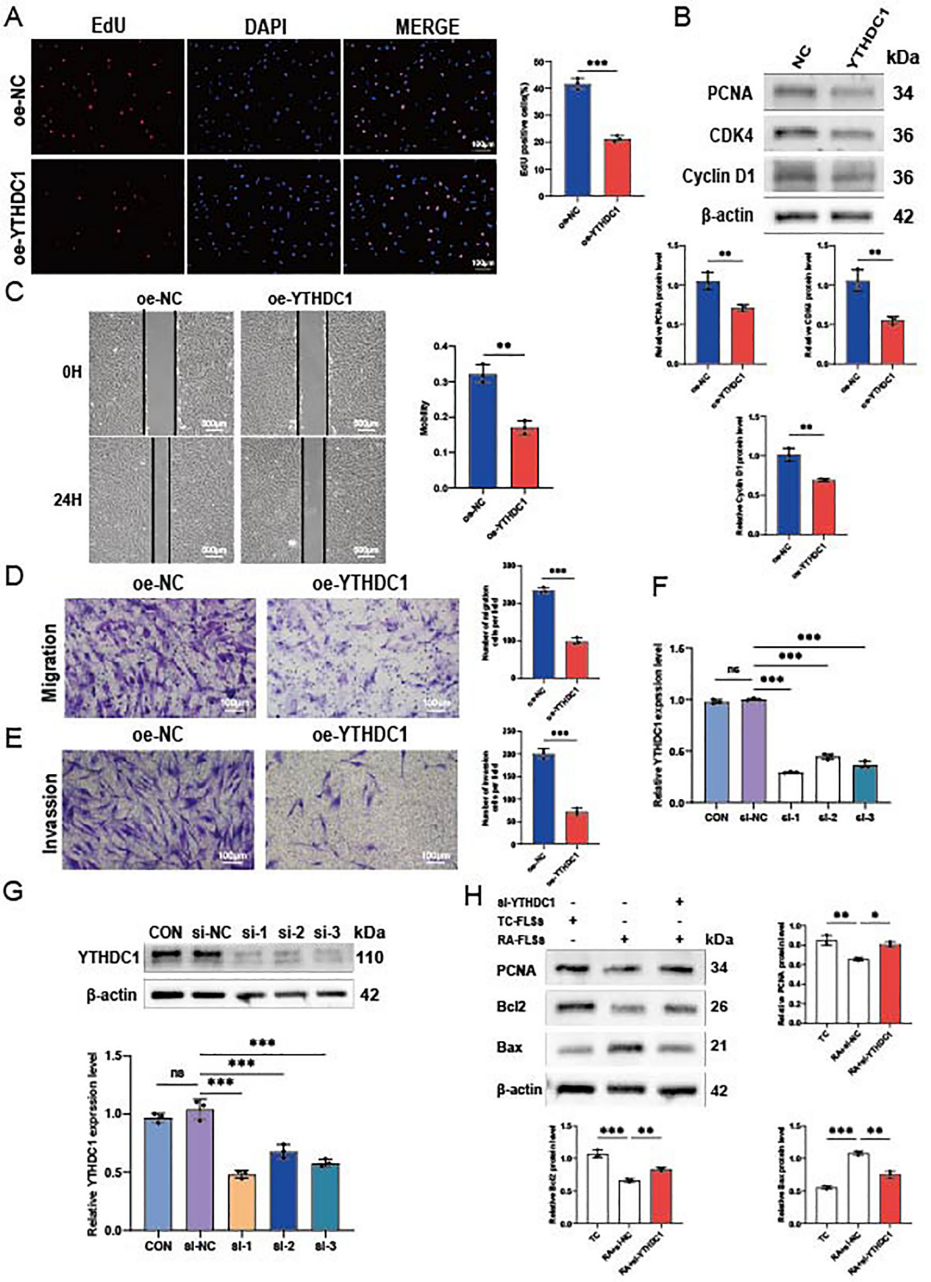
YTHDC1 influences the migration, invasion, and proliferation of RA-FLSs. **(A)** A representative photomicrograph and a column chart displaying EdU assay results (n = 3) are shown. **(B)** Western blot analysis was utilized to assess the protein levels of PCNA, CDK4, and Cyclin D1 following YTHDC1 overexpression. **(C, D)** The impact of YTHDC1 on the migratory capacity of RA-FLSs was evaluated using a **(C)** wound healing assay (n = 3) and **(D)** a Transwell migration assay (n = 3). **(E)** The invasive potential of RA-FLSs was quantified using a Matrigel-coated Transwell assay (n = 3). **(F, G)** YTHDC1 knockdown was performed in FLSs, with the efficacy confirmed via quantitative RT-PCR and Western blot analysis. **(H)** Following overexpression of YTHDC1, protein expression levels of PCNA, Bcl-2, and Bax were determined by Western blot. Data are presented as means ± SD; statistical significance denoted as ns (not significant), *P < 0.05, **P < 0.01, ***P < 0.001.

We examined the impact of YTHDC1 overexpression on migration, invasion, and proliferation of RA-FLSs. **Figure 7A** shows a significant decrease in RA-FLSs proliferation due to YTHDC1 overexpression, as evidenced by EdU assay results. This reduction correlates with the downregulation of proliferation markers PCNA, CDK4, and Cyclin D1 in YTHDC1-overexpressing RA-FLSs (**Figure 7B**). The results of scratch (**Figure 7C**) and transwell experiments (**Figure 7D**) further validate a substantial decrease in migration. Additionally, **Figure 7E** highlights a notable reduction in invasiveness. Collectively, these results confirm that YTHDC1 overexpression inhibits the migratory, invasive, and proliferative capacities of RA-FLSs.

To reinforce these findings, we employed si-RNA to knock down YTHDC1, resulting in significant increases in mRNA and protein levels (**Figures 7F, G**). Western blot analysis showed that, compared to the NC group, the si-YTHDC1 group exhibited an increase in the proliferation marker PCNA and the apoptosis-related protein Bcl2, while Bax expression decreased (**Figure 7H**). These results support the hypothesis that YTHDC1 overexpression inhibits the migration, invasion, and proliferation of RA-FLSs, and promotes their apoptosis.

## 4 Discussion

Fibroblast-like synoviocytes (FLSs) play a pivotal role in causing synovial inflammation and joint damage in rheumatoid arthritis (RA) (5, 10). Besides their resistance to apoptosis, these FLSs have the capacity to migrate and penetrate articular cartilage. Therefore, it is vital to explore the molecular mechanisms underlying the tumor-like behavior of FLSs in RA, and there is a critical urgency to identify therapeutic targets for FLSs to effectively combat RA. This study emphasizes the role of m^6^A methylation mediated by YTHDC1, a key m^6^A recognition protein. YTHDC1 downregulates the expression of PCNA, CDK4, and Cyclin D1, thereby inhibiting the proliferation of RA-FLSs. Additionally, it suppresses their migration and invasion capabilities while promoting apoptosis. Collectively, these actions contribute to reduced synovial inflammation and decelerated RA progression, affirming YTHDC1’s potential as a therapeutic target in RA.

In this study, we identified 5,714 DEGs from three RA-related gene expression profiles: GSE12021, GSE55235, and GSE55457. We analyzed these DEGs with GO and KEGG pathways to identify enriched RA-related genes. KEGG analysis revealed associations with pathways including cell proliferation, osteoclast differentiation, and T-cell and chemokine signaling. Similarly, GO analysis indicated that these DEGs are mainly involved in intercellular adhesion, T-cell activation, and cytokine receptor activity. Previous studies have demonstrated that interactions among B cells, dendritic cells, and T cells can activate the immunological responses of citrulline-containing self-proteins (1). Studies have shown that CD4+ memory T cells in the synovium regulate inflammatory responses and B-cell differentiation, exacerbating RA symptoms by producing RF or ACPA (53). Under homeostatic conditions, dendritic cells modulate the production of inflammatory cytokines and the tolerogenic T-cell response, influencing the pathology of RA (54). These findings indicate that immune cell infiltration is crucial in the pathogenesis of RA. Further immune infiltration analysis revealed significant differences between the immune cell profiles of RA synovium and normal controls. Through single-cell and PPI analysis, three hub genes were identified. Subsequent GSEA analysis of these genes demonstrated significant enrichment in cell adhesion molecule and chemokine signaling pathways in their highly expressed subgroups. Notably, YTHDC1 exhibited significant negative correlations with CD8+ T cells among various immune cells, highlighting its potential role in RA pathogenesis. Moreover, ROC analysis showed that YTHDC1, with the highest AUC value of 0.719, suggests its potential as a diagnostic marker in clinical settings.

M^6^A modification, the most prevalent internal modification in mRNA, plays a crucial role in the pathogenesis of various diseases, including osteoporosis (28, 55), cardiovascular disease (56), kidney diseases (57), immune diseases (58), and tumor microenvironment (59). Recent research has revealed abnormal m^6^A levels in the peripheral blood of patients with RA (60), however its exact role in RA remains unclear. In our study, analysis of microarray data from the GEO database demonstrated significantly lower YTHDC1 expression in the synovial tissue of RA patients compared to normal controls. Further investigation indicated reduced m^6^A levels and decreased YTHDC1 expression in the synovial tissues of RA patients. These findings imply that disrupted YTHDC1-mediated m^6^A modification in synovial tissues may contribute to the pathogenesis of RA.

We further investigated the regulatory functions of YTHDC1, a unique nuclear m^6^A reader, in RA-FLSs. YTHDC1 is predominantly located in the nucleus and plays critical roles in post-transcriptional regulation, including pre-mRNA splicing (61, 62), mRNA export (63, 64), and mRNA stabilization (65–67). Our studies demonstrate that YTHDC1 is a crucial regulator of the activation and proliferation of RA-FLSs. Overexpression of YTHDC1 reduced the migration, invasion, and proliferation of these cells, while increasing apoptosis, thereby inhibiting synovial tissue growth. Conversely, knockdown of YTHDC1 was found to enhance cell proliferation and reduce apoptosis in RA-FLSs.

The findings of this study present considerable potential for translation into clinical practice, particularly in developing novel therapeutic strategies and biomarkers for RA. Identifying YTHDC1 as a key hub gene involved in the pathogenesis of RA unveils new avenues for targeted therapy. Specifically, modulating the expression or activity of YTHDC1 may attenuate the inflammatory response in RA patients, thus offering a novel therapeutic approach. Moreover, the significant correlation between YTHDC1 expression and CD8+ T cell level suggests YTHDC1 could be a biomarker for disease progression or treatment response in RA. Given that YTHDC1 expression levels are markedly reduced in the synovial tissue of RA patients, YTHDC1 may also be explored as a potential biomarker for early diagnosis or monitoring disease activity.

Our study underscores the pivotal role of YTHDC1 in the regulation of RA-FLSs. Nevertheless, it is imperative to acknowledge several limitations that could potentially influence the interpretation of our findings. First, although the sample size utilized in our study was sufficient to demonstrate significant effects, it may not represent the broader RA patient population. This limitation could introduce selection bias, potentially impacting the generalizability of our findings. Future investigations should aim to include larger and more diverse cohorts to validate our findings across various patient populations. Second, while our study concentrated on the knockdown and overexpression of YTHDC1 in RA-FLSs, the progression of RA is driven by the intricate interplay among various cell types, including T cells, B cells, and macrophages. The omission of these cell types from our analysis constrains the comprehensiveness of our conclusions. Subsequent research should broaden the scope to incorporate these additional cell types, thereby fully elucidating the role of YTHDC1 in RA pathogenesis. Third, our investigation primarily examined the impact of YTHDC1 on the migration, invasion, proliferation, and apoptosis of RA-FLSs. Nevertheless, the potential role of extracellular YTHDC1 and interactions with unidentified binding receptors in RA remains unexplored. These uncharted factors may critically influence the disease process, necessitating further investigation.

## 5 Conclusion

**Figure 8:**
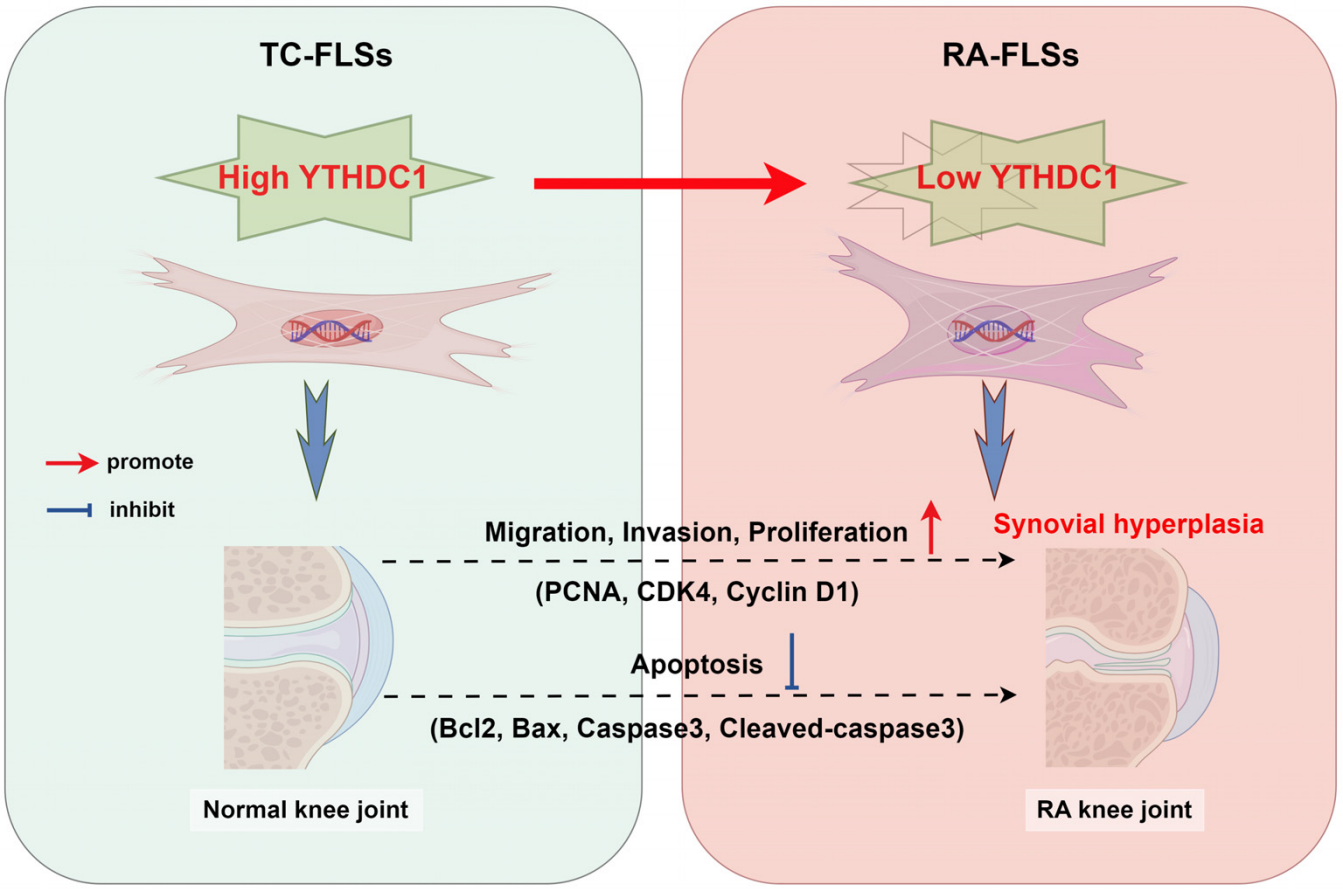
The diagram depicts the regulatory effect of YTHDC1 on the activities of Fibroblast-Like Synoviocytes (FLSs) in the context of rheumatoid arthritis (RA), encompassing their migration, invasion, proliferation, and apoptosis processes. As RA advances, hyperplasia and thickening of the synovial tissue occur. In parallel, enhanced expression of YTHDC1 mitigates FLS activity by influencing the expression of critical proteins including PCNA, CDK4, Cyclin D1, Bcl2, Bax, Caspase3, and Cleaved-caspase3.

In summary, this study is the first to confirm the impact of the m^6^A reader protein YTHDC1 on RA progression, utilizing bioinformatics and molecular biology to clarify its biological functions. Findings indicate that YTHDC1 serves a protective role in RA by inhibiting the migration, invasion, and proliferation of RA-FLSs, and by promoting their apoptosis, thus suppressing synovial tissue growth (**Figure 8**). This research provides new insights and strategies for early diagnosis and targeted therapy of RA, emphasizing potential theoretical advancements. Additionally, it facilitates the identification of novel biomarkers and therapeutic targets for RA.

## Data Availability

The datasets supporting the conclusions of this article are available in the NCBI GEO repository, accession numbers GSE12021, GSE55235, GSE55457, GSE159117. The experimental data used in this study are available from the corresponding author upon a reasonable request.
